# Big footsteps and new challenges

**DOI:** 10.1007/s12024-022-00482-5

**Published:** 2022-05-03

**Authors:** Claas T. Buschmann, Biagio Solarino, Takahito Hayashi

**Affiliations:** 1 Institute of Legal Medicine, University Hospital Schleswig-Holstein, Kiel / Lübeck, Germany; 2 Institute of Legal Medicine, University of Bari, Bari, Italy; 3 Department of Legal Medicine, Graduate School of Medical and Dental Sciences, Kagoshima University, Kagoshima, Japan

After nearly 15 years, the scientific Chief Editorial Team of *Forensic Science, Medicine and Pathology* has changed in January 2022 [1, 2].

First of all, we gratefully thank former Editor-in-Chief Roger W. Byard, former European Editor Michael Tsokos, and former North American Editor John Hunsaker III for their time and passion invested over the past 15 years in the journal — they have not only read, reviewed, and edited thousands of papers, they have also published extensively themselves and thus contributed to the reputation of the journal, too. Together with the Editorial Board, they made the journal what it is today.

Secondly, we would like to thank the whole team at Springer, who gave us three “newcomers” the opportunity to follow in these big footsteps. We will do our best.

Now brought to the second-most important journal worldwide in forensic medicine with regard to its current impact factor by the former Editorial team, *Forensic Science, Medicine and Pathology* will continue to explore all aspects of modern-day forensics. The range of topics covered will continue to include international forensic science, medicine, nursing, and pathology, as well as toxicology, human identification, mass disasters/mass war graves, profiling, imaging and forensic radiology, forensic age estimation, policing, wound assessment, child maltreatment, sexual assault, anthropology, archeology, entomology, botany, biology, veterinary pathology, medical-historical forensic research, and DNA. We will continue to insist on high scientific quality of papers in fluent and sufficiently readable English language. We pay great attention to evaluating the mega-authorship reports and ask to limit the number of self-citations, particularly if not necessary.

*Mors auxilium vitae (Death Is Help For The Living)*, and looking beyond the horizon is crucial in modern forensic medicine. Interdisciplinary questions arising in the daily autopsy routine can be addressed scientifically, and forensic medicine can contribute to walk new paths. Thus, we also welcome “outside-the-box” papers, i.e., scientific research from the interface of forensic medicine and other medical disciplines — there is a significant overlap between forensic medicine and several curative disciplines, especially after a second look [3–5]. This also applies to the interface of forensic medicine and the judicial system, i.e., legal assessment of forensic findings. As a sub-category of case reports, we have established “From The Court Room” as a brief case description to present and discuss — not necessarily extraordinary — autopsy and/or crime scene features in a specific case and their legal evaluation. What are legal consequences of our work for those affected, and where are the limits of forensic diagnostics? Where can we get better? With this new proposal, we can discuss once again the inference of the forensic publications in a trial [6]. How does the Judge determine the scientific value of the articles and the qualifications and credentials of a proposed expert witness? Even considering the differences among the legal systems worldwide, we are looking forward to submissions addressing these points.

*Forensic Science, Medicine and Pathology* will continue to present a balance of forensic research and reviews from around the world to reflect modern advances through peer-reviewed papers, short communications, meeting proceedings, new forensic textbook comments, and case reports. Furthermore, we will open the journal to answers to forensic questions that involve interfaces with other medical disciplines, especially with regard to complications arising from performed — or necessary, but omitted — medical procedures in the broadest sense. Forensic scientists are often involved in medical malpractice lawsuits, healthcare policy, and patient safety management. Therefore, the authors have the opportunity to discuss unusual adverse events, causes of medical malpractice, and the forensic medicine approach to such an interesting field of research. The dead can teach the living.

The scientific future of forensic medicine comprises not only of forensic issues, but involves interdisciplinary cooperation. We intend to be a relevant part of this future — and we can achieve this goal solely with the help of you, the authors and reviewers from all over the world!

- Priv.-Doz. Dr. med. Claas T. Buschmann, Kiel/Lübeck, Germany
- Editor-in-Chief
- Prof. Dr. Biagio Solarino, Bari, Italy
- Associate Editor
- Prof. Takahito Hayashi, Kagoshima, Japan
- Associate Editor

## Declarations

The editorial was written in compliance with Ethical Standards.
